# Increased Inducible Nitric Oxide Synthase Expression in Organs Is Associated with a Higher Severity of H5N1 Influenza Virus Infection

**DOI:** 10.1371/journal.pone.0014561

**Published:** 2011-01-19

**Authors:** Simon Burggraaf, John Bingham, Jean Payne, Wayne G. Kimpton, John W. Lowenthal, Andrew G. D. Bean

**Affiliations:** 1 Infection and Immunity, CSIRO Australian Animal Health Laboratory, Geelong, Victoria, Australia; 2 School of Veterinary Science, The University of Melbourne, Parkville, Victoria, Australia; Louisiana State University, United States of America

## Abstract

**Background:**

The mechanisms of disease severity caused by H5N1 influenza virus infection remain somewhat unclear. Studies have indicated that a high viral load and an associated hyper inflammatory immune response are influential during the onset of infection. This dysregulated inflammatory response with increased levels of free radicals, such as nitric oxide (NO), appears likely to contribute to disease severity. However, enzymes of the nitric oxide synthase (NOS) family such as the inducible form of NOS (iNOS) generate NO, which serves as a potent anti-viral molecule to combat infection in combination with acute phase proteins and cytokines. Nevertheless, excessive production of iNOS and subsequent high levels of NO during H5N1 infection may have negative effects, acting with other damaging oxidants to promote excessive inflammation or induce apoptosis.

**Methodology/Principal Findings:**

There are dramatic differences in the severity of disease between chickens and ducks following H5N1 influenza infection. Chickens show a high level of mortality and associated pathology, whilst ducks show relatively minor symptoms. It is not clear how this varying pathogenicty comes about, although it has been suggested that an overactive inflammatory immune response to infection in the chicken, compared to the duck response, may be to blame for the disparity in observed pathology. In this study, we identify and investigate iNOS gene expression in ducks and chickens during H5N1 influenza infection. Infected chickens show a marked increase in iNOS expression in a wide range of organs. Contrastingly, infected duck tissues have lower levels of tissue related iNOS expression.

**Conclusions/Significance:**

The differences in iNOS expression levels observed between chickens and ducks during H5N1 avian influenza infection may be important in the inflammatory response that contributes to the pathology. Understanding the regulation of iNOS expression and its role during H5N1 influenza infection may provide insights for the development of new therapeutic strategies in the treatment of avian influenza infection.

## Introduction

H5N1 influenza virus strains have been prevalent and highly pathogenic in gallinaceous birds, specifically chickens, causing acute systemic disease [1], [2], [3]. The severity of infection, however, varies dramatically between chickens and other avian species, such as ducks [1], [4], [5]. Infection of ducks is often asymptomatic, whereby H5N1 viruses, which are categorised as highly pathogenic in chickens, appear to display reduced clinical signs in the duck [6], [7], [8]. Furthermore, little is known about the immune response generated following H5N1 influenza infection in both chickens and ducks. Viral replication may be associated with an increased proinflammatory response [9], [10], [11], as chickens exhibit widespread viremia and an associated increase in cytokines, leading to inflammation. This suggests that H5N1 may be a potent inducer of proinflammatory cytokines, chemokines and free radicals in chickens [9], [11], [12], [13]. Therefore, a key question that remains unanswered in the field is what drives the pathogenicity associated with H5N1 influenza infection?

The free radical NO is an important messenger molecule linked to an array of immune responses [14]. NO mediates macrophage cytotoxicity and plays an antiviral role [15], [16], [17]. NO is generated by the enzyme NOS, which catalyses the biosynthesis of NO in a range of tissues. Currently, a number of distinct NOS isoforms have been characterised in mammals: the neuronal isoform (nNOS), inducible isoform (iNOS) and endothelial isoform (eNOS) [18], [19], [20]. The two constitutive forms, nNOS and eNOS, are activated by, and dependant on, changes in intracellular calcium, [21], [22] whereas iNOS is calcium independent [23]. It is this inducible NOS (iNOS) form which serves as a key molecule in combating viral infection, acting as a mediator of apoptosis and the acute phase protein response [24], . Excessive production of iNOS, however, may have negative effects reacting with other damaging oxidants and promoting inflammation [26]. Recently the iNOS gene was cloned in the chicken [27], which has allowed investigations of iNOS activity and the role of this gene in a range of tissues following H5N1 influenza virus infection of chickens. However, at present there is a paucity of information surrounding the duck iNOS gene and its role in the immune response to influenza virus infection.

To explore the role of iNOS and NO production and contrast this following the immune response of chickens and ducks to H5N1 influenza, we identified and cloned iNOS in ducks. We showed that levels of NO were elevated in both infected chickens and ducks when compared to uninfected birds. Given the severity of pathology associated with H5N1 infection varies in the organs of chickens and ducks, we examined the pathways leading to NO production at the prominent sites of infection. Our results showed that infected chickens have an earlier and more prevalent spread of iNOS in lung, spleen, caecal tonsil and liver tissue as compared to ducks. The high expression of iNOS in chicken organs may account for the more severe disease associated with H5N1 infection in this species.

## Materials and Methods

### Animals

Five-week-old Pekin ducks were purchased from Luv-a-Duck (Nhill, Victoria, Australia) and six-week-old broiler Ross chickens were purchased from Bartters (Bannockburn, Victoria, Australia). Animals were individually identified with leg bands and assigned to treatment groups. The ducks and chickens were fed with commercial grower chicken pellets, *ad libidum*. Water troughs that were deep enough for the ducks to float and splash were placed in each room. Each room also had a partially enclosed dry retreat with wood shavings for the ducks and chickens to sit on. All animal work was conducted with the approval of the CSIRO - AAHL Animal Ethics Committee. All procedures were conducted according to the guidelines of the National Health and Medical Research Council as described in the Australian code of practice for the care and use of animals for scientific purposes [28].

### Virus strains

Ducks and chickens were challenged with the Vietnamese H5N1 strain, A/Muscovy duck/Vietnam/453/2004. The virus was passed twice in chicken eggs to obtain a working stock. The working inoculum consisted of a 1∶100 dilution of infected allantoic fluid. A total volume of 0.5 mL was inoculated through an oral-intranasal route with each duck and chicken dose containing approximately 10^7.2^ median egg infectious doses (EID_50_) [29].

### Virus titration

Lung, caecal tonsil, liver and heart tissues were dissociated by bead beating and 10% w/v homogenates in phosphate-buffered saline were prepared. Homogenates were titrated in flat-bottomed 96-well micro-titre plates which were seeded with a Vero cell suspension (10^6^ cells per plate). Ten-fold dilutions of the samples were prepared and 0.1 mL of each dilution was added, as four replicates, to sequential wells of the plates. An uninfected cell control was present on each plate. The plates were incubated at 37°C in a humidified CO_2_ incubator and examined for the presence of cytopathic effect after 5 days. The lowest limit of viral detection, equivalent to a single infected well with optimal cell growth in all wells, was 10^0.75^ 50% tissue culture infectious doses per 0.1 mL (TCID_50_/0.1 mL).

### Isolation of lymphocytes and cell culture

Ducks and chickens were anesthetised by CO_2_ asphyxiation and spleens harvested. Single cell suspensions of leukocytes were prepared from individual spleens by dispersal through a 70 µm strainer into Petri dishes containing DMEM. Suspensions were layered over lymphoprep (Nicomed Pharma AS, Oslo, Norway) and centrifuged at 1500g_max_ for 15 min. Mononuclear cells at the interface were collected, washed, resuspended, and cultured in DMEM supplemented with 10% fetal bovine serum (FBS).

### Reagents

The synthetic dsRNA analog poly (I:C) (Invivogen) was prepared and stored as per manufacturer's instructions. Both lipopolysaccharides (LPS) from *E. coli* and chicken IFNγ were produced in our laboratory [30]. Nucleic acids were stored at −80°C, and cytokines were stored at 4°C.

### RNA isolation and reverse transcription

RNA was harvested using Tri-reagent (Sigma-Aldrich, St. Louis, MO) according to the manufacturer's instructions. Extracted RNA was subjected to DNase treatment using a DNase 1 (Sigma-Aldrich) and was then DNase-treated RNA was then reverse transcribed to complimentary DNA (cDNA) using a reverse transcription kit (Promega, Madison, WI).

### Cloning, 3-prime RACE sequencing

Specific iNOS oligonucleotide primers were designed around the conserved regions in sequences aligned from the human, mouse and chicken iNOS genes [18], [27], [31], [32]. A combination of primers (Table 1) were used to amplify the duck iNOS gene (GenBank accession No. FJ966247). Synthesised cDNA was made with gene specific primers in a standard PCR amplification performed using 35 cycles of 94°C for 1 min, 55°C for 1 min and 72°C for 3 min, with a further 15 min extension at 72°C following the last cycle using an Applied Biosystems DNA Thermocycler 480 (Perkin Elmer, USA). To determine the complete iNOS sequence, a 3-prime RACE (Rapid Amplifiaction of cDNA Ends) kit (Invitrogen) was used according to the manufacturer's instructions. DNA products of interest were gel purified using a gel extraction kit (Qiagen, Valencia, CA) and then ligated into pGemT-Easy (Promega) for sequence analyses. The iNOS gene was sequenced by Micromon DNA sequencing facility, Monash University (Clayton). The method used dye-terminator reactions performed on an ABI integrated Thermal Cycler and then run on an ABI 377 sequencer (Applied Biosystems).

**Table 1:** Primer sequences for RT-PCR and QRT-PCR used in this study.

Species	Primer name	Type	Sequence 5′-3′
duck	dkinosf1	RT-PCR FWD	ATGCTGTGCCCATGGCAGTTTGC
duck	dkinosr2	RT-PCR REV	TTAATTTGTGCTTGGACTGATGGG
chicken	chinosf3	RT-PCR FWD	ATGCTGTGCCCATGGCAGTTTGC
chicken	chinosr4	RT-PCR REV	GCCCGGACCAATGGGTTGCAAATC
duck/chicken	chdkinos	QRT-PCR FWD	CCACCAGGAGATGTTGAATATGTC
duck/chicken	chdkinos	QRT-PCR REP	TCCACCTGGTAGTAAAAG
duck/chicken	chdkinos	QRT-PCR REV	CCAGATGTGTGTTTTCCATGCA

### Nitrate production assay

NO production in sera was measured using a nitric oxide colorimetric assay kit (Roche cat No. 11756281001). Sera was irradiated and then filtered in a 10000 kd MWCO column (Satorious, Vivascience cat No. 13239-E). The assay was conducted as per manufacturer's instructions using a NO control with a standard curve plotted and samples were measured at a 550 nm wavelength.

### Semiquantitative RT-PCR (QRT-PCR)

The relative quantitation of gene expression following treatment was carried out on an ABI Prism 7700 sequence detection system and used the comparative threshold cycle (Ct) method to derive fold change gene expression, according to the manufacturer's instructions (Applied Biosystems, Foster City, CA). Primers and probes (Table 1) were designed using Primer express software and where possible were designed across intron:exon boundries. Probes were labeled with the reporter dye carboxyfluorescein (FAM) and the quencher tetramethyl-6-carboxyrhodamine (TAMRA). Breifly, triplicate (or more) samples were each measured in 25 µL reactions. PCR cycling was performed as follows: 95°C for 15 sec, 61°C for 30 sec and 68°C for 30 sec. Threshold values were set at a standard value (0.2) which corresponded to the midway point of the amplification plots. Relative gene expression was calculated using the mean values obtained with the arithmetic formula ΔΔCt (Applied Biosystems). Target gene Ct values were normalized to the endogenous control glyceraldehyde-3-phosphate-dehydrogenase, a house-keeping gene, to derive the ΔCt. This was compared with an untreated calibrator to derive the ΔΔCt and relative gene quantitation, or fold expression relative to the untreated control (calibrator), was derived using 2 ^–ΔΔCt^.

### Immunohistochemistry (IHC)

Chicken and duck tissues were fixed in 4% formaldehyde in neutral buffered saline. After no more than 2 days of formalin fixation, tissues were processed into paraffin wax by routine histological methods. Sections of tissues were cut onto slides and stained using an immunoperoxidase test. Sections were quenched with 10% hydrogen peroxide for 10 min and digested with 5 to 7 mg/mL proteinase K for 6–7 min. Sections were then incubated for 1 h with either rabbit serum directed against H5N1 influenza virus nucleoprotein [29] or with a rabbit polyclonal antibody directed against iNOS (Abcam ab3523). A secondary (goat anti rabbit) horseradish peroxidase-conjugated antibody (DAKO Envision) was then used for 45 min. Control sections were run following identical protocols but using an irrelevant primary mouse antibody. Sections were stained with aminoethylcarbazol substrate chromogen (DAKO Envision) for 5 to 6 min, and counterstained with Mayer's haematoxylin.

### Statistical analyses

To determine the significant differences between experimental groups, ANOVA or Mann-Whitney *U*-tests were performed using the fold change scores. All data are expressed as the mean±SEM and *p-values* were set at 0.05 (p≤0.05) unless indicated otherwise.

## Results

### Elevation of NO in the sera of chickens and ducks infected with H5N1 influenza

**Figure 1:**
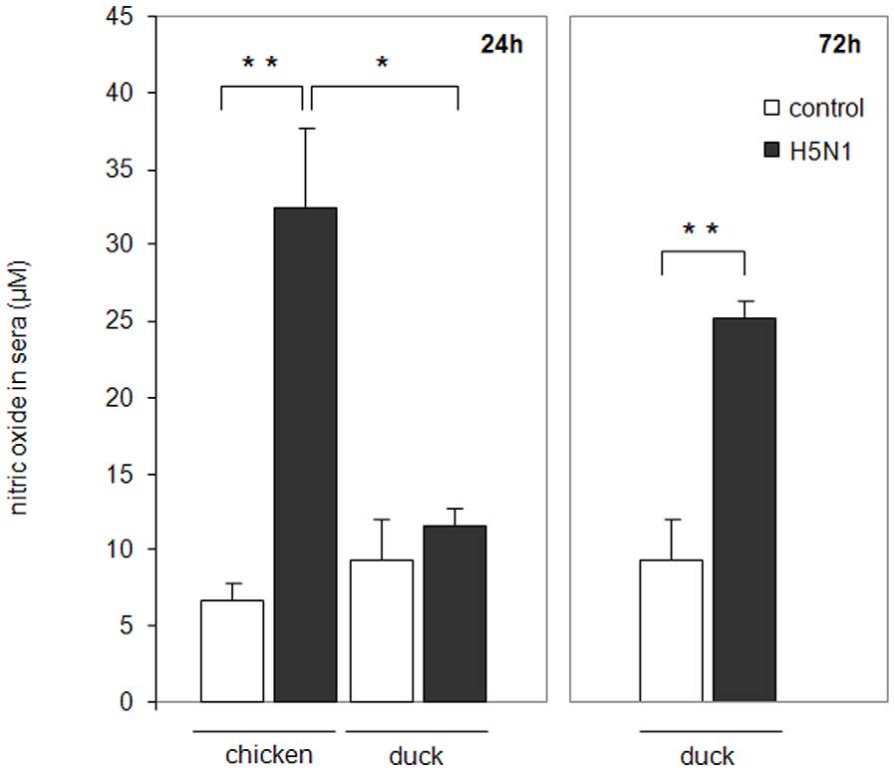
H5N1 influenza infection results in increased serum NO levels in both chickens and ducks. Serum collected from chickens and ducks infected with the H5N1 strain Muscovy/duck/Vietnam/453, as assayed for NO production during the peak of influenza infection (chickens at 24 hours, ducks at 24 and 72 hours post infection). Displayed values are the mean of 2 experiments with 4 birds in each group. A single asterisk indicates statistically significant differences of means with p<0.05, double asterisk indicates statistically significant differences of means with p<0.01.

To establish if NO expression was associated with the immune response following H5N1 influenza virus infection we assayed the concentration of this molecule in the sera of chickens and ducks. Serum was isolated from chickens at the peak of infection, 24 hours post infection (h.p.i,) whilst from ducks serum was isolated after 24 h.p.i, and 72 h.p.i, (with 72 h.p.i) being the relative peak of infection in ducks) and NO levels measured (Fig. 1). H5N1 infected chickens showed a 4-fold increase in NO in sera when compared to uninfected chickens. Ducks initially show no change in NO levels at 24 h.p.i, with H5N1 when compared to uninfected birds. After 72 h.p.i, ducks showed only a 2-fold increase in NO levels compared to uninfected ducks.

### Molecular cloning and bioinformatic analyses of duck iNOS

**Figure 2:**
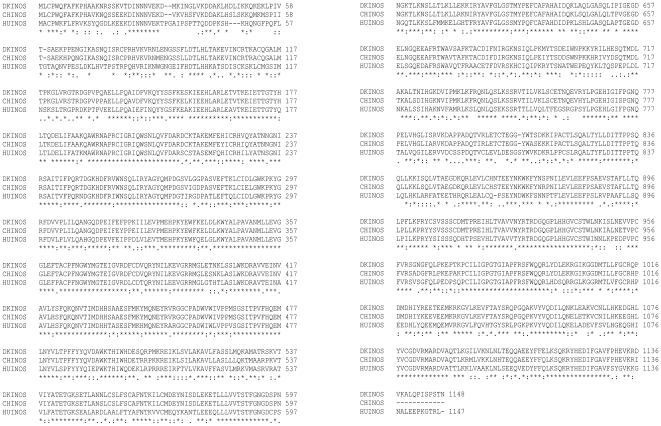
Duck iNOS amino acid sequence alignment. The open reading frame of the duck iNOS (DKINOS) sequence was analyzed through the Clustal W program and the predicted amino acid translation is shown in comparison to that of human (HUINOS) and chicken (CHINOS) iNOS. An asterisk (*) indicates identical amino acid residues while a colon (:) indicates a strongly conserved amino acid substitution and a dot (.) represents a weakly conserved amino acid substitution. Dashed sections (−) represent gaps introduced to optimize the alignment and numbers represent aa number. The nucleotide sequence was subsequently deposited to GeneBank (accession No. FJ966247).

**Figure 3:**
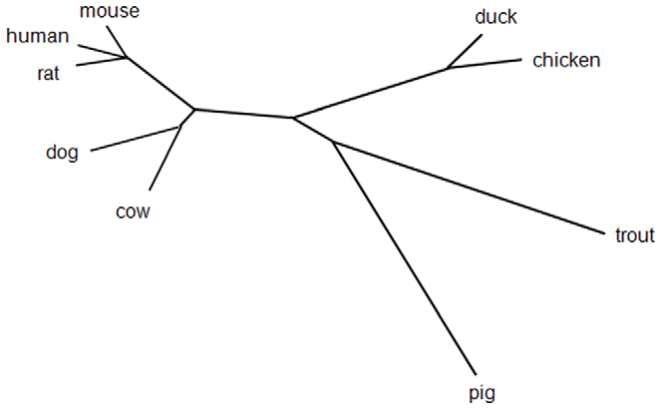
Unrooted phylogenetic tree of the iNOS gene from a range of species. An unrooted tree was constructed based on 1000 bootstrapped amino acid alignments of various iNOS members using the neighbour joining method. GeneBank accession numbers: chicken (*Gallus gallus*) NM_20496, cow (*Bos taurus*) DQ_676956, mouse (*Mus musculus*) NM_010927, dog (*Canine*) NM_001003186, rat (*Rattus novegicus*) NM_012611, human (Homo sapien) NM_000625, trout (*Oncorhynchus mykiss*) AJ_300555, pig (*Sus suscrofa*) NM_001143690.

**Figure 4:**
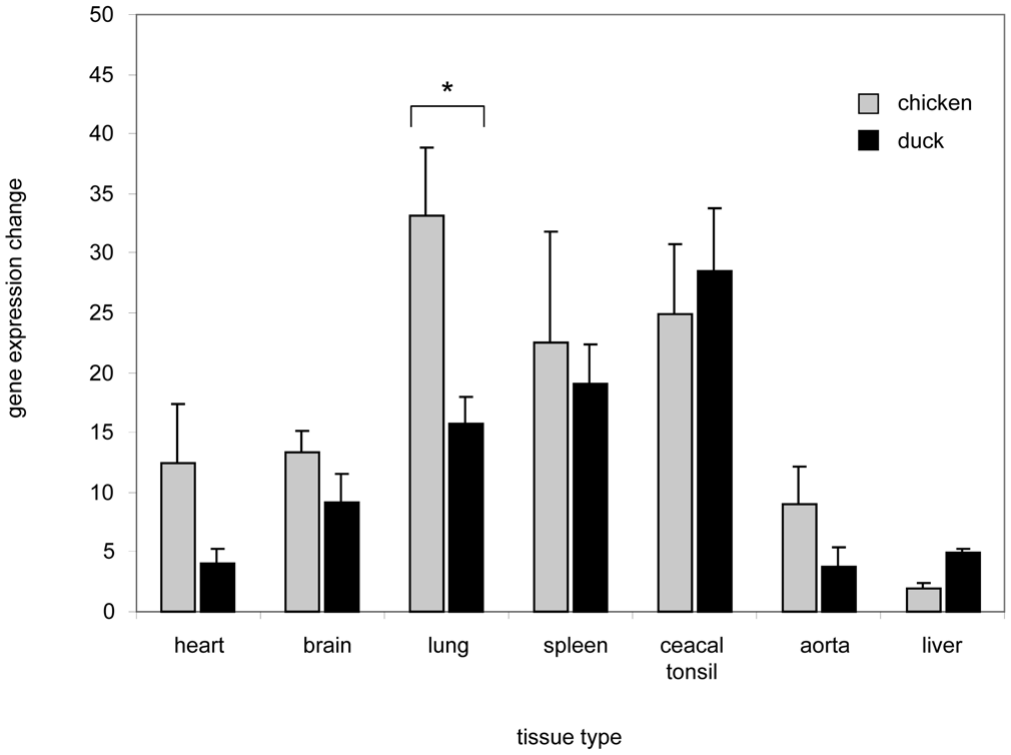
Comparison of basal iNOS levels in chicken and duck tissues measured by QRT-PCR. iNOS mRNA was measured by QRT-PCR in chicken and duck organs. GAPDH was used as a housekeeping gene to standardize results. Expression is shown as the basal fold change increase of iNOS as relative to muscle tissue. Experiments were performed in triplicate with the data representative of 3 independent experiments. An asterisk indicates statistically significant differences of means with p<0.05.

As increased levels of NO were observed in the sera of H5N1 infected animals we wanted to further characterise the levels of iNOS, an indicator of NO, in the tissues of infected animals. This is particularly relevant given the different pathogenicity witnessed following H5N1 infection in chickens and ducks. In order to determine the relationship between NO production and iNOS expression, we first had to identify iNOS in the duck. iNOS is produced as part of the oxidative burst in macrophages. Duck splenic leukocytes were therefore stimulated with LPS for 24 hours and PCR amplification with degenerative primers directed at duck iNOS was performed. A partial isoform II NOS (iNOS) cDNA product was obtained which was approximately 500 bp and showed 89% sequence identity with chiNOS cDNA. RLM 3 prime RACE (Invitrogen) was then used to capture the complete iNOS sequence which was deposited in GenBank (accession No. FJ966247). This cloned sequence of 3447 bp encoded a protein of 1148 amino acids with a predicted molecular weight of 130 kDa (Fig. 2). The deduced amino acid sequence of duck iNOS (dkiNOS) was modelled (smart.embl-heidelberg.de) and displayed conserved regions for the binding of iNOS cofactors: heme, calmodulin, FMN, FAD and NADPH. Table 2 shows that dkiNOS has a relatively high amino acid identity to iNOS protein sequences between different species (ClustalW algorithm). The dkiNOS protein was comparable (98%) to the published chiNOS protein [27]. Furthermore, phylogenetic analyses showed dkiNOS clusters near chiNOS, supporting the fact they are similar and may have a conserved function (Fig. 3). With this identification of the duck iNOS gene we then analysed the basal levels of iNOS expression in various tissues and compared these duck levels to the basal iNOS levels observed in chickens. Gene analysis by QRT-PCR showed that iNOS expression (fold increase relative to muscle iNOS levels) in ducks was generally similar to the levels observed in chickens, however, in the lung it appears that the chicken may have higher basal levels of iNOS gene expression (Fig. 4).

**Table 2:** Similarity of duck iNOS to iNOS in other species.

Organism	GenBankaccession No.	No. of aa residues	% Identicle ORF (nucleotides)	% Conserved	Total similarity %
Gallusgallus	NM_204961	1136	91.02	07.21	98.23
Bostaurus	DQ_676956	1156	68.51	22.05	90.57
Mus musculus	NM_010927	1144	65.46	24.73	90.20
Canine	NM_001003186	1154	67.15	22.35	89.51
Rattus novegicus	NM_012611	1147	65.82	23.53	89.36
Homo sapien	NM_000625	1147	65.56	23.71	89.27
Oncorthynchusmykiiss	AJ_300555	1083	59.92	27.88	87.81
Sus suscrofa	NM_001143690	1204	45.34	30.14	75.49

### iNOS is induced by mitogens in duck and chicken splenic leukocytes

**Figure 5:**
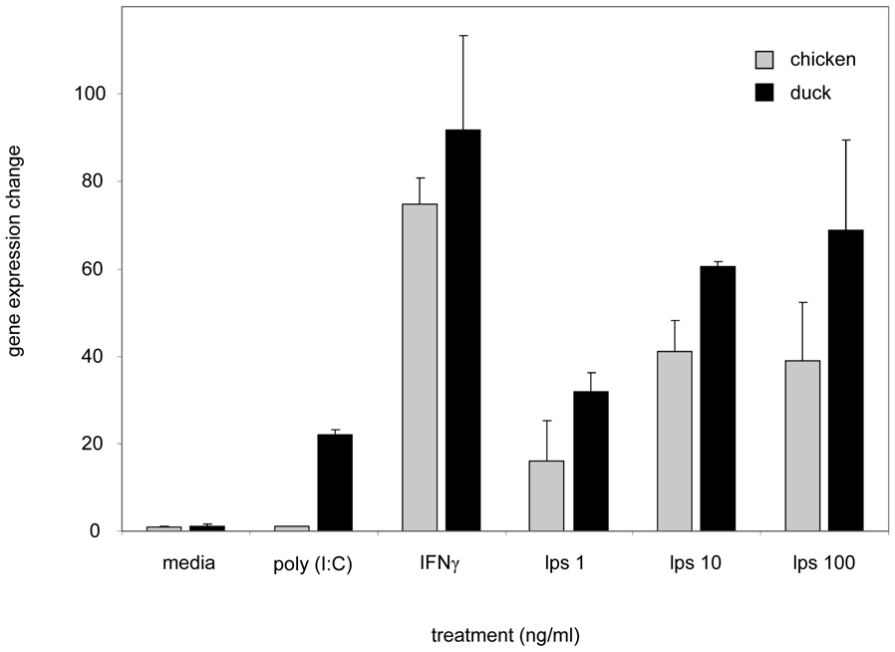
Activated chicken and duck splenocytes measured for iNOS expression levels by QRT-PCR. Chicken and duck splenocytes were cultured for 20 hours with a range of concentrations of LPS (1, 10, and 100 µg/ml) or a single concentration (10 µg/ml) of poly(I:C) or recombinant chicken IFNγ protein. RNA was collected from the cells and quantified iNOS gene levels determined. Obtained iNOS mRNA values were normalized to GAPDH and expression levels are shown as mean fold expression change relative to un-stimulated controls. Experiments were performed in triplicate and data are representative of 3 independent experiments.

**Figure 6:**
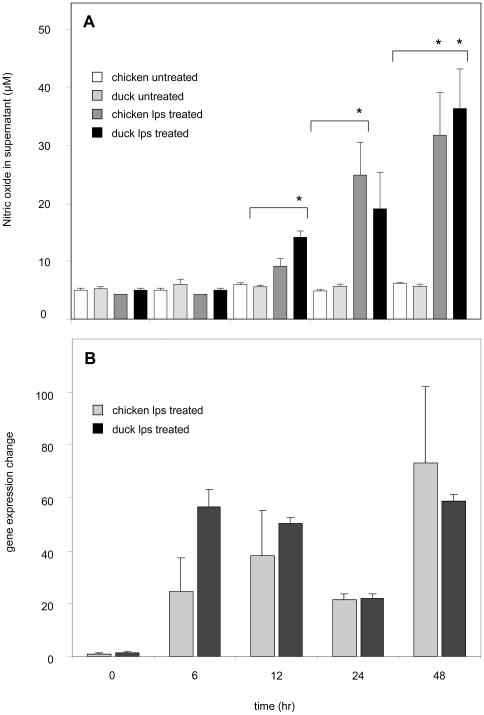
NO and iNOS levels increase in a similar fashion following LPS stimulation of chicken and duck splenocytes. Chicken and duck splenocytes were cultured over a time-course of 6–48 hours with a concentration of LPS (10 µg/mL). (**A**) NO levels, measured by a colorimetric assay, were increased in a similar fashion in chicken and duck cell culture supernatants. (**B**) iNOS gene expression levels, measured by QRT-PCR, were increased in chicken and duck splenocytes. Levels of iNOS gene expression were determined in conjunction with the house keeping gene GAPDH and expression levels are shown as mean fold expression change relative to un-stimulated splenocytes. Experiments were performed in triplicate and data are representative of 3 independent experiments. An asterisk indicates statistically significant differences of means with p<0.05.

Previous studies have shown that iNOS levels can be elevated following the addition of LPS or IFNγ in cell culture. To assess this newly identified duck iNOS gene, and compare its expression with that of chicken, splenocyte activation cultures were carried out. After 24 h of culture, iNOS levels were elevated in chicken and duck cells stimulated with IFNγ and LPS (increased 80 and 50 fold, respectively). However, the synthetic dsRNA analog poly(I:C) only increased iNOS expression in duck splenic leukocytes compared to chicken (Fig. 5). To test whether increased iNOS expression is associated with increased NO production, splenocytes were cultured with LPS for 48 hours and supernatants were tested using the colorimetric NO assay. At a range of timepoints LPS stimulated NO production in a fashion that correlated with iNOS gene induction (Fig. 6A and B).

### Chickens and ducks infected with H5N1 have increased iNOS expression

**Figure 7:**
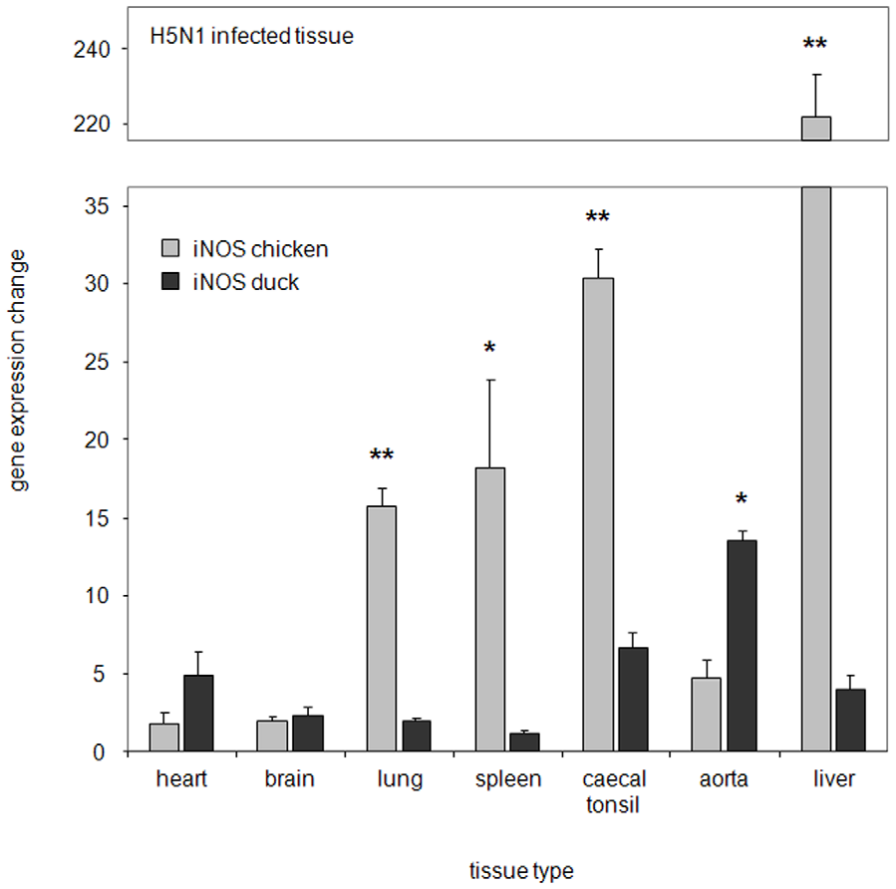
H5N1 influenza infection increases iNOS expression at higher levels in chickens compared to ducks. QRT-PCR was carried out on various tissues from chickens and ducks infected with H5N1 and compared to uninfected controls. Chicken samples were taken at 24 hours post infection, duck samples were taken at 72 hours post infection. Data represents the mean fold expression of either duck or chicken iNOS relative to each uninfected tissue type with GAPDH used as the housekeeping gene. Displayed values are the mean of 2 experiments with 4 birds in each group. Single asterisk indicates statistically significant differences of means with p<0.05, double asterisk indicates statistically significant differences of means with p<0.01.

Since we observed increases in NO in H5N1 infected sera we wanted to assess the levels of iNOS, as a potential indicator of NO, in a range of tissues from infected animals. Therefore, chickens and ducks inoculated with H5N1 influenza (Muscovy duck/Vietnam/453/2006) were sampled and iNOS levels measured at the peak of their relative infection (highest viral titres were at 24 h.p.i, for chickens and at 72 h.p.i, for ducks). The largest change in iNOS expression was observed in the liver of infected chickens (220-fold), with caecal tonsil, spleen and lung tissue also showing increased iNOS mRNA expression (30-fold, 17-fold and 15-fold, respectively). However, the heart and brain showed little significant change in iNOS mRNA expression (Fig. 7). In comparison to chickens, however, infected ducks indicated lower levels of iNOS in all organs except cardiac tissue. Ducks appeared to show an increased iNOS expression in heart tissue (5-fold), with the highest increase in aortic tissue (15-fold). Caecal tonsil tissue also showed some elevation in iNOS expression (5-fold), however, unlike chickens, lung, liver and spleen tissue showed only marginal increases compared to uninfected ducks.

### Co-localisation of iNOS and H5N1 influenza virus antigen

**Figure 8:**
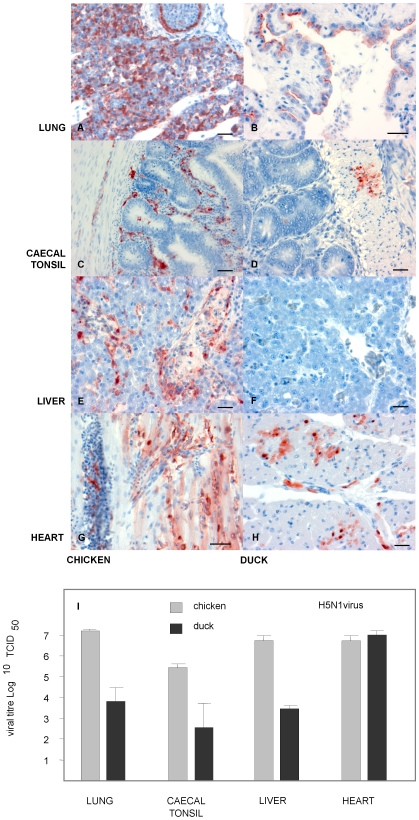
H5N1 infection appears more widespread in chicken tissues in comparison to duck tissues. IHC for H5N1 antigen, left-hand panel shows staining in chicken tissues, right-hand panel shows staining in duck tissues (**A**) Chicken lung 24 hours post infection (h.p.i), with IHC stain showing H5N1 viral antigen as red/brown colour. (**B**) Duck lung 72 h.p.i., H5N1 antigen was less prevalent in duck lung tissue than in chicken. H5N1 antigen was detected in single cells scattered within the lung parenchyma and in the hyaline cartilage. (**C**) Chicken caecal tonsil 24 h.p.i., with H5N1 in caecal lymphoid follicles and submucosa. (**D**) Duck caecal tonsil 72 h.p.i., with similar H5N1 antigen staining. (**E**) Chicken liver tissue 24 h.p.i., with severe H5N1 antigen staining. (**F**) Duck liver 72 h.p.i., showed no signs of viral antigen. (**G**) Chicken heart tissue 24 h.p.i. showed H5N1 staining in the myocardium and typically near blood vessels. (**H**) Duck heart tissue 72 h.p.i., with IHC H5N1 antigen staining the myocardium. All scale bars  = 50 µm. (**I**) The graph shows viral replication efficiency between chickens and ducks across a range of organs following H5N1 Vt453 infection. In chickens 24 h.p.i, lung, caecal tonsil, liver and heart tissue showed between 5.5 and 7 log_10_ TCID_50_. Similarly, ducks had 7 log_10_ TCID_50_ of virus in heart tissue, but moderately less virus in other tissues, between 2.5 and 4 log_10_ TCID_50_.

**Figure 9:**
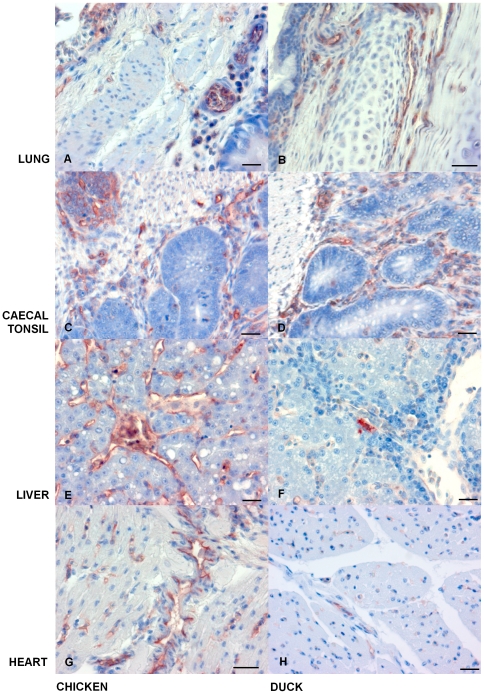
During H5N1 Influenza infection iNOS appears more highly expressed in chicken tissues when compared to duck tissues. IHC for iNOS antigen staining, with left-hand panel showing chicken tissues and right-hand panels showing duck tissues. (**A**) Chicken lung 24 h.p.i., IHC stain (red/brown) for iNOS mainly in the submucosa. (**B**) Duck lung 72 h.p.i., IHC iNOS staining in the submucosa. (**C**) Chicken caecal tonsil 24 h.p.i., with iNOS in lymphoid follicles, the submucosa and lymphoid aggregates. (**D**) Duck caecal tonsil 72 h.p.i., IHC staining of iNOS mainly in the submucosa. (**E**) Chicken liver tissue 24 h.p.i., iNOS present in the sinusoidal endothelium. (**F**) Duck liver 72 h.p.i., little or no iNOS detected. (**G**) Chicken heart 24 h.p.i., iNOS in the myocardium and proximity to blood vessels (presumably endothelial cells). (**H**) Duck heart 72 h.p.i., with low levels of IHC staining for iNOS. All scale bars  = 50 µm.

As iNOS was upregulated in H5N1 virus infected tissues we assessed the co-localisation of iNOS with regard to virus. Antibody staining for H5N1 antigen was observed in a wide range of chicken organs. In contrast, H5N1 antigen was restricted to fewer sites in infected ducks. In the chicken H5N1 was predominantly found in respiratory and intestinal tissue, spleen, liver as well as heart. However, H5N1 antigen was mainly restricted to the respiratory tract, intestinal tissue and heart of infected ducks (Fig. 8). Antibody staining for iNOS was observed to be present in tissues which were high in H5N1 antigen and was more prevalent in chickens than in ducks (Fig. 9). Uninfected chickens and ducks had little iNOS staining present. During infection with H5N1, chicken lung tissue showed iNOS staining in the respiratory epithelium and the surrounding sub mucosa. In contrast, duck lung tissue showed iNOS staining in the hyaline cartilage, with less staining present in the sub mucosa (Fig. 9A and B). In both chicken and duck caecal tonsil tissue, iNOS was prevalent in the lamina propria and in lymphocyte aggregates (Fig. 9C and D). Nevertheless, tissue from infected chicken liver showed iNOS staining in the lumen of sinusoidal areas, whilst duck liver had little iNOS staining present (Fig. 9E and F). Chicken heart tissue stained positive for iNOS in the myocardium and in endothelial cells surrounding blood vessels. Duck heart tissue had few iNOS positively stained cells present in the myocardium (Fig. 9G and H).

Uninfected tissues stained with either rabbit serum directed against H5N1 influenza virus nucleoprotein or with a rabbit polyclonal antibody directed against iNOS (Abcam ab3523), showed little to no staining in either species. Control antibody staining sections were run following identical protocols but using an irrelevant primary antibody control. To investigate the viral replication of H5N1 virus and confirm H5N1 antigen staining in chicken and duck, tissues were harvested for viral analysis. In chickens H5N1 replicated to high viral titres within 24 h.p.i (between 5–7 log_10_ TCID_50_) with lung, liver and heart tissue showing the highest viral levels. In contrast, ducks appear to have little or no viral replication 24 h.p.i (data not shown), but rather showed the highest viral replication at 72 h.p.i. Duck heart tissue showed the highest viral replication levels (7 log_10_ TCID_50_), whilst lung, liver and caecal tonsil had lower viral replication levels.

## Discussion

Continued sporadic outbreaks of H5N1 influenza virus infection highlights the need for alternative strategies to deal with infection. Moreover, without a greater understanding of host-pathogen interactions, the management of H5N1 will continue to be difficult. Nitric oxide is produced primarily as an effector molecule as part of the hosts defence response [33]. However, it has been postulated that the outcomes of this defence response may be either beneficial or detrimental to the host depending on the level of NO produced [12], [24]. Therefore, understanding the role that NO and iNOS play during H5N1 infection in chickens and ducks [1], [4], [34] may provide insights into the underlying mechanisms and differences observed in disease severity. To investigate this we identified the presence of the iNOS gene in ducks and confirmed that dkiNOS acts in a similar fashion to its chicken counterpart [27]. Furthermore, we describe the changes in iNOS levels in H5N1 infected chicken tissues and compared these to iNOS levels in infected duck tissue. Tissue specific increases in iNOS production in the chicken appear to be associated with a higher degree of disease severity in H5N1 infection in chickens when compared to ducks. However, although we have observed this association for the Vt453 H5N1 strain, since there is some degree in pathogenicity between the various isolates of H5N1 virus, there may also be varying degrees of association of iNOS with pathogenicity.

NO is known to be a key molecule in the immune response against viral infection [35], [36]. Studies of human and mouse iNOS and the associated NO production, have reported that these molecules are often active in concert with IFNγ, to protect against viral infection [37], [38], [39], however, their role in avian species is less defined. Moreover, it has been shown that upregulation of IFNγ and the induction of other proinflammatory molecules increase the production of NO [38], [40]. The demonstration that dkiNOS expression is increased in a similar fashion to chiNOS, when stimulated by inflammatory mitogens, such as LPS and IFNγ (Fig. 5 and Fig. 6), suggests a comparable role in the innate immune response of avian species. As dkiNOS is structurally similar to other iNOS homologues (Fig. 2) and appears to be associated with NO production, it may be expected that the iNOS response in ducks is similarly conserved. Furthermore, given the growing evidence for the antiviral activity of iNOS in the chicken [41], particularly following influenza infection [34], [42], it may be further expected that dkiNOS has the ability to be upregulated during the course of H5N1 influenza virus infection.

It is still unclear what effect iNOS and other proinflammatory molecules have during influenza infection. IFNγ has been shown to upregulate iNOS expression [40] and likewise we observed that the addition of IFNγ to duck cells increased both iNOS mRNA expression and NO production. Correspondingly, H5N1 influenza studies in mammals have shown that IFNγ levels are increased and similarly H5N1 infected chickens have both elevated levels of IFNγ and other cytokines [11], [37], [43]. H5N1 viruses are often associated with an increase in inflammatory molecules [44], supporting a possible role for iNOS [34]. With this in mind, we examined NO and iNOS expression during H5N1 infection in chickens and ducks. In H5N1 infected chickens we observed that iNOS expression was increased in the lung, spleen and caecal tonsil by as much as 30-fold. This widespread increase in iNOS may be due to the fact that in chickens H5N1 strains tend to have a broad tissue tropism [8], [44], [45]. Viral analysis showed high viral titres in chicken lung, caecal tonsil, liver and heart tissue. In chickens, the respiratory system appears to be the predominant site of infection, however, virus quickly spreads to other organs, such as the heart and liver, with the infection becoming systemic [46]. This may be crucial in the manifestation of excessive levels of NO during infection.

Intriguingly, the levels of iNOS expression were increased over 200-fold in the liver. Basal levels of iNOS gene expression are relatively low in chicken liver tissue compared to chicken lung, spleen and caecal tonsil (Fig. 4) and this may partly account for its high fold increase in this organ. Nevertheless, the high levels of iNOS observed in chicken liver tissue may also be associated with the observed production of acute phase proteins [25]. An acute phase response is involved in many inflammatory infections [13], [44], [47] whereby proteins, such as serum amyloid A (SAA), have been associated with NO production from macrophages [25]. Furthermore, during studies investigating the acute phase response following H5N1 influenza, we have observed increased levels of SAA in chickens, whilst the levels of SAA in H5N1 infected ducks was comparably low (Burggraaf *et al*., manuscript in preparation). This higher level of iNOS production in tissues infiltrated with virus raises the question of the role iNOS regulation has in the outcome of viral infections. In chickens, high iNOS production may have an impact on the severity of the pathology witnessed during H5N1 infection.

In contrast to the chicken, H5N1 infection in ducks is characterised by a predilection for viral replication in muscle and lung tissue [29]. We observed the highest viral levels in the heart with less viral replication occurring in the lung, liver and caecal tonsil tissue. We identified that heart and aorta tissue in ducks had the greatest increases in iNOS expression, although IHC for iNOS in duck heart tissue was less prevalent than in infected chickens. Levels of iNOS expression were also increased in the caecal tonsil of ducks and IHC for iNOS expression showed its localisation in the proximity of H5N1 antigen in the lamina propria. This increase in iNOS could be associated with an upregulated inflammatory response during infection. It has been reported that H5N1 infection of duck heart muscle may cause myocarditis and similarly infection of the brain may cause encephalitis [29]. The resultant inflammatory responses in these organs may contribute to some of the observed morbidity in infected ducks. Levels of iNOS may potentially become increased at sites of inflammation, suggesting that in a similar fashion to the chicken, increases in duck iNOS may be associated influenza infection. This may be due to either the action of genes that iNOS stimulates, such as acute phase proteins and cytokines or the actual production of NO and its potential to contribute to apoptosis [24], [48]. Nevertheless, NO has the potential to generate free radicals which may damage cells [12] and therefore it is important that NO and iNOS be tightly regulated [49]. Kacergius *et al*., (2006) indicated that during influenza infection, increases in NO synthesis through iNOS gene induction, contributed to lung damage. For these reasons, iNOS has been implicated in the pathology associated with some viral models [34], [35], [50]. The question still remains as to the relevance of the association of iNOS and NO in the observed pathology of H5N1 influenza infection. At present there is a clear association, however, the role iNOS plays in viral pathogenicity for each of the species is yet to be determined. Future work will be directed at determining whether the pathological outcomes are a result of iNOS expression and are the cause of disease severity.

Although both H5N1 infected chickens and ducks have increased circulating levels of NO in comparison to uninfected animals, the levels of iNOS gene expression and tissue expression observed by QRT-PCR and IHC are markedly different between the two species. IHC staining for iNOS in chicken H5N1 infected liver tissue showed its presence predominantly in the sinusoidal areas (presumably endothelial cells), whilst in caecal tonsil and lung tissue iNOS was also observed in lymphoid aggregates and in endothelial cells around blood vessels. In comparison to infected chickens, IHC for duck iNOS appears restricted to the caecal toncil, with infrequent staining in upper respiratory and heart tissue. Furthermore, no iNOS staining was found in infected duck liver tissue. This suggests that iNOS may contribute to higher NO production within H5N1 infected chicken organs than in infected duck organs. However, comparably higher NO levels in the chicken may be only a part of the problem for the associated increased severity of H5N1 infection. It is likely other immune genes such, as viral recognition receptors and inflammatory cytokines have a role in the varied pathogenicity following H5N1 influenza infection. For example, Barber *et al*., (2010), demonstrated that chickens lack the viral sensing receptor RIG-I which when expressed in chicken cells improves the response to H5N1 influenza infection [51]. Furthermore, studies by Okada *et al*., (2009) suggested that the response to H5N1 influenza in chickens could be improved following reduction of the inflammatory immune cascade [52]. Therefore, it would appear that further research may be required to fully elucidate the mechanisms behind the varied immune response between chickens and ducks.

In conclusion, it may be postulated that since H5N1 viruses are often associated with an increased pathogenicity in chickens, it may be that iNOS expression in chickens becomes dysregulated and this may impact on disease severity through the overproduction of free radicals and the resultant damage to cellular function. With this in mind, further investigation into the inflammatory response during H5N1 viral infection is needed. A better understanding of the inflammatory response following H5N1 influenza infection may help in developing new strategies and approaches for modulating the outcomes of these infections in chickens and ducks.
